# Real-World Use of Androgen-Deprivation Therapy: Intensification Among Older Canadian Men With de Novo Metastatic Prostate Cancer

**DOI:** 10.1093/jncics/pkab082

**Published:** 2021-10-01

**Authors:** Christopher J D Wallis, Shawn Malone, Ilias Cagiannos, Scott C Morgan, Robert J Hamilton, Naveen S Basappa, Cristiano Ferrario, Geoffrey T Gotto, Ricardo Fernandes, Tamim Niazi, Krista L Noonan, Fred Saad, Sebastien J Hotte, Huong Hew, Katherine F Y Chan, Laura Park Wyllie, Bobby Shayegan

**Affiliations:** 1 Department of Urologic Surgery, Vanderbilt University Medical Center, Nashville, TN, USA; 2 Division of Radiation Oncology, The Ottawa Hospital, University of Ottawa, Ottawa, ON, Canada; 3 Division of Urology, The Ottawa Hospital, University of Ottawa, Ottawa, ON, Canada; 4 Department of Surgery, University of Toronto, Princess Margaret Cancer Centre, Toronto, ON, Canada; 5 Department of Oncology, Cross Cancer Institute, University of Alberta, Edmonton, AB, Canada; 6 Department of Oncology, McGill University, Segal Cancer Centre, Jewish General Hospital, Montreal, QC, Canada; 7 Department of Surgery, Southern Alberta Institute of Urology, University of Calgary, Calgary, AB, Canada; 8 Division of Medical Oncology, London Regional Cancer Program, London, ON, Canada; 9 Radiation Oncology Department, Jewish General Hospital, McGill University, Montreal, QC, Canada; 10 BC Cancer Agency, University of British Columbia, Surrey, BC, Canada; 11 Genitourinary Oncology, Centre Hospitalier de l’Université de Montréal, University of Montreal, Montréal, QC, Canada; 12 Department of Oncology, McMaster University, Juravinski Cancer Centre, Hamilton, ON, Canada; 13 Medical Affairs, Janssen Inc, Toronto, ON, Canada; 14 Institute of Urology, St Joseph’s Healthcare, McMaster University, Hamilton, ON, Canada

## Abstract

**Background:**

Despite the wealth of evidence demonstrating the efficacy of treatment intensification beyond androgen-deprivation therapy (ADT) among patients with de novo metastatic castration-sensitive prostate cancer (mCSPC), little is known of its real-world use. This study examined the real-world uptake of ADT treatment intensification among older men in a large Canadian province.

**Methods:**

We performed a retrospective population-based cohort study using province-wide linked administrative data in Ontario, Canada. Patients 66 years of age and older with de novo mCSPC were included and their treatment with conventional ADT-based regimens, ADT plus next-generation androgen receptor axis–targeted therapy, and ADT plus docetaxel were identified and stratified by time.

**Results:**

From 2014 to 2019, 3556 patients were identified with de novo mCSPC. Most patients (n = 2794 [78.6%]) were treated with a conventional ADT regimen, whereas 399 (11.2%) patients received ADT intensification with docetaxel and 52 (1.5%) patients received abiraterone acetate plus prednisone. In a time-stratified analysis of ADT intensification before and after the pivotal AA+P trial (LATITUDE), AA+P uptake increased from 0.5% to 3.0%, whereas docetaxel use dropped from 12.0% to 10.0%. The median survival of the study population was 18 months (interquartile range = 10-31).

**Conclusions:**

The majority of patients with de novo mCSPC are treated with ADT alone in the Canadian real-world setting, despite randomized clinical trial evidence of benefit with the use of ADT-intensified regimens. As ADT treatment intensification is substantially underused, better understanding of the barriers to treatment and targeted education to address them are needed.

Prostate cancer (PCa) is the most common cancer among Canadian men (1-3). For patients with de novo and recurrent metastatic PCa, androgen-deprivation therapy (ADT) has been a mainstay of care since the 1940s (4). In patients with metastatic castration-sensitive PCa (mCSPC), the effectiveness of ADT treatment is modest: Progression to castration resistance is inevitable, with the reported median overall survival (OS) ranging from 17 months (5) to 3-3.5 years (6) depending on patient age and data source (ie, real world vs clinical trials). Recently, the CHAARTED (4) and STAMPEDE (arms C and E) (7,8) trials have shown a statistically and clinically significant benefit associated with adding docetaxel to ADT (ADT intensification) in patients with high-volume disease. The LATITUDE (9) and STAMPEDE (arm G) (10) studies have also demonstrated improved survival with the addition of abiraterone acetate and prednisone (AA+P) to ADT among high-risk patients, whereas prostate radiation therapy added to ADT improves OS in patients with low-burden disease (11). Furthermore, treatment options for mCSPC in Canada were recently enriched by the addition of 2 more androgen receptor axis–targeted (ARAT) therapies—namely, apalutamide (12) and enzalutamide (13). Overall, the therapeutic importance of intensifying conventional ADT therapy by adding systemic therapies (ARAT or docetaxel) has been recognized and incorporated into clinical practice guidelines in Canada and elsewhere (14,15).

Among men with metastatic PCa, those diagnosed with de novo or synchronous disease have worse outcomes than those diagnosed with metachronous disease (6,16). Approximately 60% of patients with newly diagnosed mCSPC have high-volume disease (11), and ADT intensification among patients with high-risk or high-volume mCSPC has been an established standard of care for several years. Little is known, however, about the real-world uptake of ADT intensification among patients with newly diagnosed mCSPC and the effect of geographic variation on the uptake of ADT intensification.

The primary objective of this study was to examine the real-world treatment use patterns in patients with de novo mCSPC over time. Secondarily, we explored the geographic variation in uptake of ADT intensification.

## Methods

### Study Design

A retrospective population-based cohort study was conducted using provincewide linked administrative data in Ontario, Canada. The study received ethics approval from Advarra institutional review board (Pro00037601).

### Setting

Canada has a universal, publicly funded health-care system for all legal and permanent residents. Ontario is the most populous province in Canada, with almost 14.7 million residents, representing about 40% of the Canadian population (17). The provincial health system provides universal hospital and physician services that are free at the point of care for residents. The costs of outpatient prescription drugs are publicly covered for patients older than 65 years and for those who are younger than 24 years of age and not covered by a private plan (18).

The province is divided into 14 local health integration networks (LHINs) that are responsible for local health-care planning and delivery (19). In Ontario, cancer care services are coordinated by Cancer Care Ontario, a government agency responsible for collecting cancer data, developing clinical standards, and planning cancer services (20). Of approximately 100 community and academic hospitals that deliver inpatient and outpatient cancer care services in the province, 14 hospitals are designated as regional cancer centers that service each LHIN.

### Data Sources

The patient-level dataset was created using health administrative databases housed at ICES (www.ices.on.ca), an independent, nonprofit research corporation funded by the Ministry of Health and Long-Term Care. These databases contain publicly funded administrative health service records for the Ontario population eligible for health coverage. They are linked using encrypted patient-specific identifiers. Supplementary Table 1 (available online) presents a description of databases used to create the study dataset. Under section 45(1) of the Personal Health Information Protection Act, 2004, ICES has statutory authority to conduct health services research without consent using anonymized administrative data; therefore, patient consent was waived.

### Study Population

A cohort of patients with de novo mCSPC in the province was defined as follows: male patients aged 66 years and older who had metastatic disease at the time of diagnosis with PCa (index date) between January 1, 2014, and March 31, 2020. Patients were excluded if they were female sex, aged 65 years or younger, had missing or invalid identification numbers, were not eligible for provincial health insurance coverage 2 years before diagnosis, or had missing data for key exposure for analyses. The Ontario Drug Benefit coverage starts at 65 years of age; therefore, we included patients aged 66 years or older to capture at least 1 year of full prescription data.

During this time period, AA was not publicly reimbursed for mCSPC, and patients received AA through compassionate use programs. Because AA is prescribed concurrently with prednisone, however, which is publicly funded, we captured patients who were receiving AA+P by creating a proxy for AA+P using the following criteria: We first identified patients prescribed continuous prednisone 5 to 10 mg for 3 months or more (overlapping prescriptions with no more than 14 days elapsing between prescriptions). We then excluded patients who consulted with a rheumatologist in the 12 months before cohort entry/index date to ensure that they did not receive prednisone for reasons other than the care of mCSPC. Finally, we excluded any patients who had had a prescription for prednisone for longer than 3 months in the year preceding cohort entry. We validated this approach by applying the proxy criteria to a population of patients with metastatic castration-resistant PCa (mCRPC) identified from the same province-linked databases. In patients with mCRPC, AA is publicly reimbursed; thus, the proxy definition could be tested within this population to determine the test characteristics of our proxy criteria. Among 991 patients with mCRPC, the validation exercise demonstrated that the prednisone proxy definition had 100% specificity, 87.7% sensitivity, 100% positive predictive value, and 85.3% negative predictive value for the use of AA (Supplementary Tables 2-4, available online).

### Treatment Approaches

Patients were categorized into 1 of 4 cohorts according to their treatment patterns. Conventional ADT (cohort 1) included patients on ADT alone, an antiandrogen alone, ADT plus an antiandrogen for 3 months or less (flare protection), or ADT plus an antiandrogen for more than 3 months (combined androgen blockade). ADT intensification regimens were represented by ADT with or without an antiandrogen plus AA+P (cohort 2) and ADT with or without an antiandrogen plus docetaxel (cohort 3). Patients who received none of these treatment approaches were compiled in cohort 4 (non-ADT). Supplementary Table 5 (available online) provides a full definition for each cohort.

### Variables

The study population was described at baseline using variables, including patient sociodemographic characteristics (age, socioeconomic status, LHIN, rurality), PCa attributes (prostate-specific antigen at diagnosis, Gleason score), health status (Charlson Comorbidity Index [CCI]; history of key conditions other than PCa, including diabetes, myocardial infarction, congestive heart failure, liver or kidney disease), and health-care use (number of visits to a general practitioner, status of a long-term care resident, history of hospitalizations). Details on each variable are presented in Supplementary Table 6 (available online). As ICES databases do not contain imaging data, we could not classify patients by disease volume or risk.

### Study Endpoints

Primary end points included: 1) the proportion (%) of patients with de novo mCSPC across the treatment patterns stratified by time (pre- and post-LATITUDE) and 2) the presence of regional variation in the uptake of ADT intensification.

As a secondary endpoint, we estimated the OS (median, interquartile range [IQR]) of patients with de novo mCSPC from their index date, which was defined as the time of diagnosis for metastatic disease. Patients without events (ie, death) were censored at their last date of follow-up. Mortality was defined as death from any cause.

### Statistical Analysis

Descriptive statistics were summarized for baseline patient characteristics across the cohorts. Characteristics of patients across the treatment patterns were compared using χ^2^ and *t* test statistics for categorical and continuous variables, respectively.

To quantify the effect of regional variation across LHINs, multivariable logistic regression was performed, with the ADT intensification vs conventional ADT used as the dependent outcome. The model included the following baseline characteristics as covariates: age, socioeconomic status, CCI score, hospitalization event, long-term care residency, history of diabetes, myocardial infarction, cerebrovascular accident, congestive heart failure, chronic obstructive pulmonary disease, hypertension, arrhythmia, dementia, liver disease, kidney disease, Gleason score, number of general practitioner visits, and prostate-specific antigen values at diagnosis. OS was calculated using the Kaplan-Meier method. All statistical analyses were conducted using SAS software, version 9.4 (SAS Institute Inc, Cary, NC, USA). All statistical tests were 2-sided, and *P* < .05 was considered statistically significant.

## Results

### Patient and Disease Characteristics

From 2014 to 2019, we identified 3556 patients who presented with de novo metastatic PCa. The baseline characteristics are summarized in Table 1. Over the 5-year period, 2794 (78.6%) patients were treated with a conventional ADT regimen for mCSPC, whereas 399 (11.2%) patients received ADT intensification with docetaxel therapy and 52 (1.5%) patients received AA+P for mCSPC. Patients who were treated with docetaxel were younger (mean [SD] = 72.6 [4.7] years of age) and healthier (mean [SD] CCI score = 0.15 [0.7]) than those across the other treatment patterns. Patients in the AA+P group had the highest CCI score (mean [SD] = 0.67 [1.62]), and the greatest proportion of those were from rural areas (19.2%). There was no statistically significant difference in socioeconomic status across the treatment patterns. The median prostate-specific antigen at diagnosis was lower among patients who received conventional ADT (88 ng/mL [IQR = 26-346]) compared with ADT intensification regimens (121 ng/mL [IQR = 19-485] and 152 ng/mL [IQR = 37-451] for the AA+P and docetaxel cohorts, respectively).

**Table 1.** Baseline characteristics, by treatment cohort

Patient demographics	Conventional ADT	ADT + AA+P	ADT + docetaxel	Non-ADT	*P* ^a^
Total, No. (%)	2794 (78.6)	52 (1.5)	399 (11.2)	311 (8.7)	
Mean (SD) age, y	78.31 (7.39)	76.71 (7.26)	72.57 (4.82)	76.82 (8.64)	<.001
SES, No. (%)					
Quintile 1	595 (21.3)	9 (17.3)	61 (15.3)	64 (20.6)	.08
Quintile 2	556 (19.9)	10 (19.2)	70 (17.5)	54 (17.4)	
Quintile 3	552 (19.8)	13 (25.0)	74 (18.5)	61 (19.6)	
Quintile 4	539 (19.3)	14 (26.9)	97 (24.3)	68 (21.9)	
Quintile 5	552 (19.8)	6 (11.5)	97 (24.3)	64 (20.6)	
Rurality, No. (%)					
Nonrural	2,479 (88.7)	42 (80.8)	361 (90.5)	274 (88.1)	<.001
Rural	315 (11.3)	10 (19.2)	38 (9.5)	37 (11.9)	
Medical care and comorbidity					
CCI score, mean (SD)	0.35 (1.00)	0.67 (1.62)	0.15 (0.72)	0.36 (0.98)	<.001
No. of GP visits, mean (SD)	9.38 (8.43)	8.63 (6.81)	7.84 (5.70)	9.73 (11.62)	<.001
Hospitalizations, No. (%)	482 (17.3)	11 (21.2)	42 (10.5)	58 (18.6)	.02
Ever an LTC resident, No. (%)	39 (1.4)	1-5^b^	0 (0.0)	10 (3.2)	.002
Diabetes, No. (%)	194 (6.9)	3-7^b^	33 (8.3)	28 (9.0)	.27
History of MI, No. (%)	67 (2.4)	0 (0.0)	12 (3.0)	4-8^b^	.86
History of CVA, No. (%)	60 (2.1)	0 (0.0)	1-5^b^	6 (1.9)	.12
History of CHF, No. (%)	219 (7.8)	1-5^b^	13 (3.3)	16 (5.1)	.004
History of COPD, No. (%)	175 (6.3)	1-5^b^	19 (4.8)	15 (4.8)	.47
History of hypertension, No. (%)	289 (10.3)	1-5^b^	40 (10.0)	30 (9.6)	.47
History of arrhythmia, No. (%)	47 (1.7)	0 (0.0)	1-5^b^	1-5^b^	.27
History of dementia, No. (%)	262 (9.4)	0 (0.0)	10 (2.5)	47 (15.1)	<.001
History of liver disease, No. (%)	28 (1.0)	0 (0.0)	7 (1.8)	1-5^b^	.22
History of kidney disease, No. (%)	264 (9.4)	1-5^b^	17 (4.3)	26 (8.4)	.05
PCa characteristics					
PSA at diagnosis (3 mo)					
PSA test, No. (%)	2,243 (80.3)	34 (65.4)	352 (88.2)	146 (46.9)	<.001
Median (IQR)	88 (26-346)	121 (19-485)	152 (37-451)	12 (8-33)	<.001
Biopsy Gleason score, No. (%)					
<7	7 (0.3)	0 (0.0)	1-5^b^	1-5^b^	<.001
7	198 (7.1)	0 (0.0)	24-28^b^	77-81^b^	
>7	1150 (41.2)	9 (17.3)	207 (51.9)	60 (19.3)	
Unknown	1439 (51.5)	43 (82.7)	165 (41.4)	169 (54.3)	

a χ^2^ and *t* test statistics were used for categorical and continuous variables, respectively; tests of statistical significance were 2-sided. AA+P = abiraterone acetate plus prednisone; ADT = androgen-deprivation therapy; CCI = Charlson Comorbidity Index; CHF = congestive heart failure; COPD = chronic obstructive pulmonary disease; CVA = cerebrovascular accident; GP = general practitioner; IQR = interquartile range; LTC = long-term care; MI = myocardial infarction; PCa = prostate cancer; PSA = prostate-specific antigen; SES = socioeconomic status.

b Denotes cases where, to protect confidentiality, a range of patients involved has been provided to avoid the potential for patient identification.

### Primary and Secondary Endpoints

As shown in Table 1, 451 (12.7%) patients received ADT intensification. Among them, the majority received docetaxel (88.5% [n = 399]).

Table 2 presents a time-stratified analysis representing the uptake of ADT intensification regimens before and after the pivotal 2017 LATITUDE (9) study, which demonstrated the efficacy of AA+P on OS. Whereas AA+P prescriptions increased from 0.5% to 3% in the pre- vs post-LATITUDE period, respectively, docetaxel treatment dropped from 12% to 10%.

**Table 2.** Androgen-deprivation therapy, by time period of prostate cancer diagnosis

Time period of PCa diagnosis	Conventional ADT^a^, No. (%)	ADT + AA+P, No. (%)	ADT + docetaxel, No. (%)	Non-ADT, No. (%)	*P* ^b^
Before June 3, 2017	1679 (77.7)	10 (0.5)	260 (12.0)	212 (9.8)	<.001
After June 3, 2017	1115 (79.9)	42 (3.0)	139 (10.0)	99 (7.1)	–

a Conventional ADT cohort included patients with the following treatment patterns: ADT alone, antiandrogen alone, ADT plus an antiandrogen for 3 months or less, or ADT plus an antiandrogen for more than 3 months. AA+P = abiraterone acetate plus prednisone; ADT = androgen-deprivation therapy; PCa = prostate cancer.

b Multivariable logistic regression analysis with a 2-sided *P* value was used.

Table 3 shows the distribution of patients in the de novo mCSPC cohort across the LHINs, by treatment. The unadjusted uptake of ADT intensification ranged from 8.5% (LHIN “G”) to 20.9% (LHIN “B”), with *P* *<* .001. When adjusted for baseline characteristics, the regional variation in the uptake of ADT intensification compared with conventional therapies remained statistically significant (*P *=* *.003).

**Table 3.** Distribution of patients across treatment groups, by local health integration network

Ontario LHIN (deidentified)	Conventional ADT^a^, No. (%)	ADT intensification^b^, No. (%)	Non-ADT, No. (%)	All LHINs, No. (%)	*P* ^c^
A	220 (70.7)	53 (17.0)	38 (12.2)	311 (100.0)	<.001
B	130 (71.4)	38 (20.9)	14 (7.7)	182 (100.0)	
C	81 (75.7)	19 (17.8)	7 (6.5)	107 (100.0)	
D	145 (75.9)	22 (11.5)	24 (12.6)	191 (100.0)	
E	216 (78.0)	32 (11.6)	29 (10.5)	277 (100.0)	
F	267 (78.3)	41 (12.0)	33 (9.7)	341 (100.0)	
G	186 (78.8)	20 (8.5)	30 (12.7)	236 (100.0)	
H	334 (79.1)	58 (13.7)	30 (7.1)	422 (100.0)	
I	160 (79.2)	28 (13.9)	14 (6.9)	202 (100.0)	
J	186 (80.5)	29 (12.6)	16 (6.9)	231 (100.0)	
K	284 (80.5)	43 (12.2)	26 (7.4)	353 (100.0)	
L	326 (80.7)	45 (11.1)	33 (8.2)	404 (100.0)	
M	179 (86.1)	14-18^d^	11-15^d^	208 (100.0)	
N	80 (87.9)	5-9^d^	2-6^d^	91 (100.0)	
All LHINs	2794 (78.6)	451 (12.7)	311 (8.7)	3556 (100.0)	

a Conventional ADT cohort included patients with the following treatment patterns: ADT alone, antiandrogen alone, ADT plus an antiandrogen for 3 months or less, or ADT plus an antiandrogen for more than 3 months. AA+P = abiraterone acetate plus prednisone; ADT = androgen-deprivation therapy; LHIN = local health integration network.

b ADT intensification included ADT plus AA+P and ADT plus docetaxel.

c Multivariable logistic regression analysis with a 2-sided *P* value was used.

d Denotes cases where, to protect confidentiality, a range of patients involved has been provided to avoid the potential for patient identification.

At a median follow-up of 22.9 months, 1891 (53.2%) patients had died (data not shown). The median survival of the entire population of patients with de novo mCSPC was 18 months (IQR = 10-31).

## Discussion

Our study examined the real-world uptake of ADT intensification regimens among patients with de novo mCSPC in Ontario, Canada. We selected the time frame for our study to encompass the period beginning from the CHAARTED (4) results to the period following the LATITUDE (9) results. Although we were not able to identify the proportion of patients with high-volume or high-risk disease within our cohort, we anticipated that the real-world uptake could approach approximately 60%, mirroring the 60% of high-volume disease reported among the population with de novo mCSPC in STAMPEDE arm H (11). Even in the post-LATITUDE period, however, the proportion of patients with ADT intensification observed in our study was far lower than the proportion of patients with high-burden disease, as estimated from STAMPEDE arm H.

Despite the preponderance of level 1 evidence that ADT intensification leads to longer survival, most patients in our study population received conventional ADT. This finding is suggestive of a statistically significant degree of underutilization of these life-prolonging therapies in an otherwise eligible population. The observed underutilization of ADT intensification in our study is consistent with the results of a large retrospective study using Veterans Health Administration claims data in the United States (21). This study, which examined a shorter time period (4 years) and smaller sample population (approximately 1500 patients) than our study, also reported a strong pattern of underutilization (21). Approximately 14% of patients received an ADT intensification treatment: 8% received docetaxel and 6% AA+P. In recently presented data at the American Society of Clinical Oncology 2021 Annual meeting, similar results were found in analyses of patients treated in a variety of contexts in the United States using the Optum health insurance claims database, a Medicare database, and the ConcertAI Oncology dataset. In STAMPEDE arm H, examining the role of radiation therapy to the prostate in patients with advanced disease (11), Parker et al. reported that 18% of patients received docetaxel in addition to ADT as part of standard care.

We conducted a time-stratified analysis to examine treatment patterns before and after the LATITUDE (9) results were presented. Although the uptake of AA+P increased following LATITUDE, the magnitude of the growth was unexpectedly low for an oral medication that could be prescribed in a broader population, including chemotherapy-eligible and chemotherapy-ineligible patients.

The study also demonstrated statistically significant intraprovincial variation in the use of ADT intensification. The presence of geographic disparity in the use of life-prolonging therapies after adjusting for common confounding factors is concerning because the Canadian health-care system advocates for equal access to essential treatments regardless of geographical location. Additionally, cancer care in Ontario has a reputation of a world-leading system that is well organized and adequately funded. Given similar findings from previous research (22,23), a more detailed exploration into the possible reasons of regional variation is required.

The OS rate for the entire cohort was substantially lower than what was reported in clinical trials. As the majority of Ontario men were treated with conventional ADT, we were unable to thoroughly assess the impact of intensified treatment (ie, with ARAT or docetaxel) on survival. Yet, when compared with the control arm of CHAARTED, where patients were treated with ADT alone, the median OS was 47.2 months in the overall population and 34.4 months for those with high-volume disease (24). Similarly, in STAMPEDE, the median OS of the CHAARTED-based high-volume subgroup was higher compared with our observations (8,25). On the contrary, a US-based real-world study using the National Cancer Institute’s Surveillance, Epidemiology, and End Results (SEER) database between 2004 and 2014 reported a median OS of 17 to 23 months among those 65 years of age and older(5), which is in line with the OS estimate from our study population of older patients, although the average age of patients enrolled in the clinical trials was 66 to 68 years. Therefore, age appears to have a substantial impact on the mortality of patients with de novo mCSPC and shows that outcomes in the real-world setting can vary from those reported in the clinical trial setting.

As with any other study, this study has its limitations. First, we used a proxy to identify patients who received AA+P. Although this approach was validated, the patient identification algorithm may have missed some patients on AA+P, resulting in the lower capture of AA+P in our study. The sensitivity of the proxy criteria based on our own validation study was 87.7%; therefore, assuming that the rate of detection is similar for mCSPC and mCRPC, we could have missed about 12% of patients who were receiving AA+P. We may have also observed an underestimation of the AA+P rate because our dataset captured treatment patterns only up to March 2020, and more recent data could be expected to show further uptake of AA+P. Second, the lack of data granularity (eg, lack of imaging data) did not enable us to examine heterogeneity in outcomes by disease volume or risk. Because we could not differentiate patients by disease volume or risk, our study cannot fully estimate the magnitude of treatment underintensification in the studied population. It is possible that the actual incidence of high-risk or high-volume disease in our mCSPC cohort was either closer to 60% (as seen in other population-based studies and clinical trials) or was closer to 13% (ie, the rate of treatment intensification we observed). Third, our study cohort consisted of older patients, with the median age being 77 years, whereas the average age of patients enrolled in the de novo mCSPC trials was 66 to 68 years. This difference in age is indicative of the poor representativeness of the clinical trials because the aforementioned SEER-based study observed three-quarters of its patient population to be 65 years of age and older (5). Another Canadian real-world study of AA+P use in mCRPC also had patients with a median age of 77 years (26). Finally, patients on mCSPC therapies that were not approved nor publicly funded in Canada during our study period (ie, enrolled in clinical trials or the use of newer ARAT therapies, such as apalutamide and enzalutamide) were not captured. Given these limitations, a more definitive conclusion as to whether an underestimation exists will depend on observations from other provinces across Canada. An evaluation of the rates and patterns of uptake in treatment intensification for the newer ARAT therapies that can be prescribed in mCSPC regardless of volume or risk criteria can also contribute to decision making.

Despite strong evidence to support improved survival and quality of life among patients with de novo mCSPC who are receiving systemic therapy beyond ADT, ADT treatment intensification is substantially underused. Suboptimal uptake of life-prolonging therapies in the real world may translate to poorer health outcomes for patients. Conventional ADT alone is no longer considered sufficient for the majority of patients. The new standard of care for mCSPC is treatment intensification of ADT, with additional systemic therapy where a wider use of ARAT therapies is supported regardless of risk or volume criteria. Further efforts are needed to educate health-care providers on the management of de novo mCSPC. Evidence of regional variation in the uptake of combination therapy needs further study.

## Funding

This study was sponsored by Janssen Inc.

## Notes

**Role of the funder:** Janssen supported the conduct of the study and facilitated the management and analysis of the data but had no role in the decision to submit the manuscript for publication. H. Hew, K. F. Y. Chan, and L. Park-Wyllie are employees of Janssen Inc. No other authors received payment for the development of the manuscript.

**Disclosures: CJDW:** Consulting or advisory role: (Janssen Oncology). **SM—**Honoraria (Astellas Pharma, Bayer, Janssen, Sanofi); travel, accommodations, expenses (Sanofi, TerSera). **IC—**Honoraria (AbbVie, Ferring); travel, accommodations, expenses (TerSera). **SCM—**Consulting or advisory role (Astellas Pharma, Bayer, Janssen, TerSera). **RJH—**Honoraria (AbbVie, Amgen, Astellas Pharma, Bayer, Janssen); research funding (Bayer, Janssen); travel, accommodations, expenses (Janssen). **NSB—**Honoraria (Astellas Pharma, Eisai, Ipsen, Janssen, Merck, Pfizer); consulting or advisory role (Astellas Pharma, AstraZeneca, Bayer, Bristol-Myers Squibb, Eisai, Ipsen, Janssen, Merck, Pfizer, Roche Canada); travel, accommodations, expenses (Eisai). **CF—**Honoraria (Astellas Pharma, AstraZeneca, Bayer, Lilly, Merck, Novartis, Pfizer); consulting or advisory role (AstraZeneca, Odonate Therapeutics, Pfizer, Roche); speakers’ bureau (Janssen); research funding (Astellas Pharma, AstraZeneca, Immunomedics, Janssen, Merck, Novartis, Pfizer, Roche, Sanofi, Seattle Genetics). **GTG—**Honoraria (Amgen, Astellas Pharma, Bayer, Janssen, Merck); consulting or advisory role (Amgen, Astellas Pharma, Bayer, Janssen, Merck); expert Testimony (Janssen); travel, accommodations, expenses (Janssen). **RF—**Honoraria (Bayer, Merck, Pfizer); consulting or advisory role (Bayer, Janssen, Novartis Canada Pharmaceuticals Inc, Pfizer); travel, accommodations, expenses (Janssen). **TN—**Honoraria (AbbVie, Astellas Pharma, Bayer, Janssen Oncology, Sanofi, TerSera); consulting or advisory role (AbbVie, Bayer, Janssen Oncology, Sanofi, TerSera); research funding (Astellas Pharma, Bayer, Sanofi, TerSera); travel, accommodations, expenses (Janssen Oncology, Sanofi, TerSera). **KN—**Consulting or advisory role (AstraZeneca, Bristol-Myers Squibb, Ipsen, Janssen Oncology, Merck, Pfizer, Roche Canada, Sanofi); speakers’ bureau (Merck). **FS—**Honoraria (AbbVie, Astellas Pharma, AstraZeneca, Bayer, Janssen Oncology, Myovant Sciences, Pfizer, Sanofi); consulting or advisory role (AbbVie, Astellas Pharma, AstraZeneca/MedImmune, Bayer, Janssen Oncology, Myovant Sciences, Pfizer, Sanofi); research funding (Astellas Pharma, AstraZeneca, Bayer, Bristol-Myers Squibb, Janssen Oncology, Pfizer, Sanofi). **SJH—**Honoraria (Astellas Scientific and Medical Affairs Inc, Bayer, Janssen Oncology, Merck); consulting or advisory role (Astellas Pharma, AstraZeneca, Bayer, Bristol-Myers Squibb, Eisai, Ipsen, Janssen, Merck, Pfizer, Roche Canada); research funding (Astellas Pharma, AstraZeneca, Ayala Pharmaceuticals, Bristol-Myers Squibb, Clovis Oncology, Janssen Oncology, Merck, Pfizer, Roche/Genentech, Seattle Genetics, SignalChem); travel, accommodations, expenses (Eisai). **HH—**Employee of Janssen. **KFYC—**Employee of Janssen. **LPW—**Employee of Janssen. **BS—**Honoraria (AbbVie, Astellas Pharma, Bayer, Janssen, Knight Pharmaceuticals, TerSera); consulting or advisory role (AbbVie, Astellas Pharma, Bayer, Janssen, Knight Pharmaceuticals, TerSera); speakers’ bureau (AbbVie, Astellas Pharma, Bayer, Janssen, Knight Pharmaceuticals, TerSera); research funding (Astellas Pharma, Janssen, Merck).

**Author contributions: CJDW:** conceptualization, methodology, validation, formal analysis, investigation, data curation, writing—original draft, review and editing, visualization. **SM**: conceptualization, methodology, validation, formal analysis, investigation, writing—original draft, review and editing, supervision. **IC:** conceptualization, methodology, formal analysis, visualization, writing—review and editing. **SCM:** conceptualization, methodology, formal analysis, visualization, writing—review and editing. **RJH:** conceptualization, methodology, formal analysis, visualization, writing—review and editing. **NSB:** conceptualization, methodology, formal analysis, visualization, writing—review and editing. **CF:** conceptualization, methodology, formal analysis, visualization, writing—review and editing. **GTG:** conceptualization, methodology, formal analysis, visualization, writing—review and editing. **RF:** conceptualization, methodology, formal analysis, visualization, writing—review and editing. **TN:** conceptualization, methodology, formal analysis, visualization, writing—review and editing. **KN:** conceptualization, methodology, formal analysis, visualization, writing—review and editing. **FS:** conceptualization, methodology, formal analysis, supervision, writing—review and editing. **SJH:** conceptualization, methodology, formal analysis, visualization, writing—review and editing. **HH:** conceptualization, formal analysis, project administration, funding acquisition, writing—review and editing. **KFYC:** conceptualization, methodology, investigation, formal analysis, data curation, visualization, project administration, writing—original draft, review and editing. **LPW:** conceptualization, methodology, validation, data curation, formal analysis, investigation, project administration, funding acquisition, writing—original draft, review and editing. **BS**: conceptualization, methodology, validation, formal analysis, investigation, writing—original draft, review and editing, supervision.

**Acknowledgements:** IQVIA team was hired by Janssen Inc to assist with writing and editing the manuscript. Initial ideas, study concepts, analyzed data, its interpretation as well as the clinical significance were provided to IQVIA by its authors and Janssen Inc.

Dr Wallis had full access to all the data in the study and takes responsibility for the integrity of the data and the accuracy of the data analysis. This study contracted ICES Data & Analytic Services (DAS) and used deidentified data from the ICES Data Repository, which is managed by ICES with support from its funders and partners: Canada’s Strategy for Patient-Oriented Research (SPOR), the Ontario SPOR Support Unit, the Canadian Institutes of Health Research, and the Government of Ontario. Parts of this material are based on data and information provided by Cancer Care Ontario (CCO). Parts of this material are based on data and information compiled and provided by CIHI.

**Disclaimers:** The opinions, results and conclusions reported are those of the authors. No endorsement by ICES or any of its funders or partners is intended or should be inferred. The opinions, results, view, and conclusions reported in this paper are those of the authors and do not necessarily reflect those of CCO. No endorsement by CCO is intended or should be inferred. The analyses, conclusions, opinions and statements expressed herein are those of the author, and not necessarily those of CIHI.

**Prior presentation:** Some of the content had been presented in abstract form at American Society of Clinical Oncology Genitourinary 2021.

## Data Availability

Health administrative databases for Ontario, Canada, are housed at ICES (www.ices.on.ca).

## Supplementary Material

pkab082_Supplementary_Data

## Abbreviations

AA+P: Abiraterone acetate plus prednisone
ADT: Androgen Deprivation Therapy
ARAT: Androgen Receptor-Axis-Targeted Therapy
CHF: congestive heart failure
COPD: Chronic obstructive pulmonary disease
GP: general practitioner
LHIN: Local Health Integration Networks
LTC: long-term care
mCSPC: Metastatic Castration-Sensitive Prostate Cancer
MI: myocardial infarction
PCa: Prostate Cancer
PSA: Prostate Specific Antigen
SEER: Surveillance, Epidemiology, and End Results
SES: Socio-Economic Status
OS: Overall Survival
