# Supplementary material for: Real-World Use of Androgen-Deprivation Therapy: Intensification Among Older Canadian Men With de Novo Metastatic Prostate Cancer
Source: JNCI Cancer Spectr. 2021 Oct 1;5(6):pkab082. doi: 10.1093/jncics/pkab082 (PMC8678925; doi:10.1093/jncics/pkab082)
Supplement: pkab082_Supplementary_Data [file pkab082_supplementary_data.pdf]

Supplementary Material

Supplementary Table 1. Description of databases that informed the development of the study dataset

Database	Description
Ontario Cancer Registry (OCR)	The OCR is collected by Cancer Care Ontario and contains information on all Ontario residents who have been newly diagnosed with cancer ("incidence") or who have died of cancer ("mortality"). All new cases of cancer are registered, except non-melanoma skin cancer.
Registered Person Database (RPDB)	The RPDB provides basic demographic information (age, sex, location of residence, date of birth, and date of death for deceased individuals) for those issued an Ontario health insurance number. The RPDB also indicates the time periods for which an individual was eligible to receive publicly funded health insurance benefits and the best-known postal code for each registrant on July 1st of each year.
Continuing Care Reporting System (for Chronic Care) (CCRS)	The CCRS database is compiled by the Canadian Institute for Health Information and contains demographic, clinical, functional, and resource utilization information for individuals receiving facility-based continuing care (also known as extended, auxiliary, or complex chronic care) in Ontario hospitals and residential care providing 24 hour nursing services (i.e. nursing home). Clinical assessment data (on the physical, functional, cognitive, and social domains of health) is ascertained using the Resident Assessment Instrument Minimum Data Set (RAI-MDS) version 2.0 which is administered by trained healthcare professionals.
Ontario Congestive Heart Failure dataset (CHF)	The Ontario Congestive Heart Failure Database is an ICES-derived cohort that was created using a definition of ≥2 physician billing claims with a diagnosis of CHF (OHIP diagnosis code: 428) and/or ≥1 inpatient hospitalization or same day surgery record with a diagnosis of CHF (ICD-9 diagnosis code: 428; ICD-10 diagnosis code: I50; in the primary diagnostic code space) in a two-year period applied to hospitalization (DAD), same day surgery (SDS), and physician billing claims (OHIP) data to determine the diagnosis date for incident cases of CHF in Ontario.
Ontario Chronic Obstructive Pulmonary Disease Dataset (COPD)	The Ontario COPD Database is an ICES-derived cohort that is created using two separate algorithms applied to inpatient hospitalization (DAD), same day surgery (SDS) records, and physician billing claims (OHIP) data to determine the diagnosis date for incident cases of COPD in Ontario. In an algorithm which maximizes sensitivity, the definition for COPD is any physician billing claim with a diagnosis for COPD (OHIP diagnosis codes: 491, 492, 496) or any inpatient hospitalization or same day surgery record with a diagnosis for COPD (ICD-9 diagnosis codes: 491,

	492, 496; ICD-10 diagnosis codes: J41- J44; in any diagnostic code space).
Discharge Abstract Database (DAD)	The DAD is compiled by the Canadian Institute for Health Information and contains administrative, clinical (diagnoses and procedures/interventions), demographic, and administrative information for all admissions to acute care hospitals, rehab, chronic, and day surgery institutions in Ontario. At ICES, consecutive DAD records are linked together to form 'episodes of care' among the hospitals to which patients have been transferred after their initial admission.
Ontario Hypertension Dataset (HYPER)	The Ontario Hypertension Database is an ICES-derived cohort and created using a definition of ≥2 physician billing claims with a diagnosis of hypertension (OHIP diagnosis codes: 401-405) and/or ≥1 inpatient hospitalization or same day surgery record with a diagnosis of hypertension (ICD-9 diagnosis codes: 401-405; ICD-10 diagnosis codes: I10-I13, I15; in any diagnostic code space) in a two-year period applied to hospitalization (DAD), same day surgery (SDS), and physician billing claims (OHIP) data to determine the diagnosis date for incident cases of hypertension in Ontario. Physician claims and hospitalizations with a diagnosis of hypertension occurring within 120 prior to and 180 days after a gestational hospitalization record are excluded.
National Ambulatory Care Reporting System (NACRS)	The NACRS is compiled by the Canadian Institute for Health Information and contains administrative, clinical (diagnoses and procedures), demographic, and administrative information for all patient visits made to hospital- and community-based ambulatory care centres (emergency departments, day surgery units, hemodialysis units, and cancer care clinics). At ICES, NACRS records are linked with other data sources (DAD, OMHRS) to identify transitions to other care settings, such as inpatient acute care or psychiatric care.
Ontario Diabetes Dataset (ODD)	The Ontario Diabetes Database is an ICES-derived cohort and is created using algorithms applied to inpatient hospitalization (DAD) records, same day surgery (SDS) records, and physician billing claims (OHIP) data to determine the diagnosis date for incident cases of diabetes in Ontario. For adults aged 19 years and greater, the definition for diabetes is 2 physician billing claims with a diagnosis for diabetes (OHIP diagnosis code: 250) or 1 inpatient hospitalization or same day surgery record with a diagnosis for diabetes (ICD-9 diagnosis code: 250; ICD-10 diagnosis codes: E10, E11, E13, E14; in any diagnostic code space) within a 2 year period. Physician claims and hospitalizations with a diagnosis of diabetes occurring within 120 prior to and 180 days after a gestational hospitalization record were excluded.
Ontario Health Insurance Plan Claims Database (OHIP)	OHIP claims data received by ICES contains most claims paid for by the Ontario Health Insurance Plan. The data cover all health care providers who can claim under OHIP

	(this includes physicians, groups, laboratories, and out-of-province providers. Approximately 95% of specialists and 50% of primary care physicians receive the majority of their income from fee-for-service (FFS). However, all physicians, with the exception of the few hundred family physicians who work in Community Health Centers, are required to submit shadow billings for their non-FFS services. A shadow-billing claim is identical to an FFS claim except that the payment amount is $0.00. Shadow-billings have an explain code of ‘I2’. Physicians are often provided with cash incentives to encourage them to shadow-bill. Requiring physicians to shadow-bill helps to ensure that the OHIP data accurately (more-or-less) reflects the utilization of physician services in Ontario.
Ontario Myocardial Infarction Dataset (OMID)	The Ontario Myocardial Infarction Database is an ICES-derived cohort and contains records of all inpatient hospital admissions for acute MIs (ICD-9 diagnosis code: 410; ICD-10 diagnosis code: I21; in the primary diagnostic code space) in Ontario since 1991. These admissions are ascertained using the DAD and exclude in-hospital events and admissions where there had been a previous discharge for acute myocardial infarction in the previous year. This cohort of patients with acute MI hospital admissions is linked with hospitalization (DAD), same day surgery (SDS), and physician billing claims data (OHIP) to create indicators of hospital readmission after discharge and receipt of cardiac procedures during and after the initial hospital admission.
Ontario Laboratories Information System (OLIS)	Starting in 2012 all Public Health Ontario laboratories joined OLIS. As of August 2016, OLIS has completed connections with additional hospital laboratories in 13 out of 14 LHINs. As of February 2017, Hamilton Health Sciences is now fully contributing lab results into OLIS. The OLIS library at ICES consists of 3 distinct datasets: 1. Lab orders: contains the order-level information ,including patient demographics, and provider information; 2. Test requests: contains the test ID code (called Test Request Code) and specimen information, in addition to ordering, performing and reporting facilities among other variables; 3. Observations: contains the test result information, including the result ID code (called Observation Code), values and units
New Drug Funding Program (NDFP)	The New Drug Funding Program (NDFP) is one of four publicly funded drug programs under the Ontario Public Drug Programs (OPDP). Administered by Cancer Care Ontario, the NDFP funds new, and often very expensive, cancer drugs. The program was created in 1995 to ensure that Ontario patients have equal access to high-quality intravenous (IV) cancer drugs.
Cancer Activity Level Reporting (ALR)	Activity Level Reporting (ALR) data represents the basic set of data elements required to produce the quality, cost and performance indicators for the cancer system. The data elements constitute patient level activity within the

	cancer system focused on radiation and systemic therapy services and outpatient oncology clinic visits. This data is also a key component of the Ontario Cancer Registry (OCR), which registers every malignant neoplasm diagnosed in Ontario.

Supplementary Table 2. Results of internal validation of the proxy measure of abiraterone prescription[a]

Description	No. of Patients
mCRPC cohort	991
Including those who used prednisone on or after mCRPC diagnosis date but before June 01, 2017	767
Including those who used prednisone (<01Jun2017) and abiraterone (<01Jun20117) at the same time (prescription date + supply days within +/- 7 days of the 2 drugs)	578
Including those who used prednisone 5-10 mg/day continuously ≥ 3 months with ≤ 14-day allowable gap and the prednisone initiation was on or after PCa diagnosis date	507
Total	507

[a] mCRPC = metastatic castrate-resistant prostate cancer; PCa = prostate cancer.

Supplementary Table 3. Internal validation with prednisone use as the proxy measure of abiraterone prescription[a]

Prednisone Use	Abiraterone Use, No. of Patients		Total No. of Patients
	Yes	No	
Yes	507	0	507
No	71	413	484
Total	578	413	991

[a] mCRPC = metastatic castrate-resistant prostate cancer;

Supplementary Table 4. Sensitivity and specificity of internal validation

Abiraterone (gold standard) and Prednisone used at the same time	Calculation	Results[a], %
Sensitivity (% abiraterone patients that were identified from the prednisone process)	507/507+71	87.7
Specificity	413/0+413	100.0
Positive Predictive Value (% prednisone patients that were actually on abiraterone)	507/507+0	100.0
Negative Predictive Value	413/413+71	85.3

[a]Five hundred seventy-eight patients were identified to have used prednisone at the same time as abiraterone acetate, of which 507 patients used low dose prednisone continuously after prostate cancer diagnosis. Therefore, the use of prednisone as a proxy for abiraterone acetate usage yielded a sensitivity of 87.7% and a specificity of 100%.

Supplementary Table 5. Definition and programming of treatment patterns

Approach	Pattern	Drug Use	Step	Description
Conventional ADT	1	LHRH alone	1	Including those who initiated LHRH between 60 days prior to prostate cancer diagnosis and 180 days following diagnosis
			2	Excluding those who initiated anti-androgen between 60 days prior to prostate cancer diagnosis and 180 days following diagnosis
			3	Excluding those in patterns 3-6
			4	Re-classified those who had surgical orchiectomy between 60 days prior to prostate cancer diagnosis and 180 days following diagnosis in other patterns into pattern 1
	2	Anti-androgen alone	1	Including those who initiated anti-androgen between 60 days prior to prostate cancer diagnosis and 180 days following diagnosis
			2	Excluding those who initiated LHRH between 60 days prior to prostate cancer diagnosis and 180 days following diagnosis
	3	LHRH + anti-androgen ≤ 3 months without prednisone or docetaxel	1	Including those who used both LHRH and anti-androgen between 60 days prior to prostate cancer diagnosis and 180 days following diagnosis (any drug might be initiated first)
			2	Including those who had continuous use of anti-androgen ≤ 3 months (with ≤14-day gap)
			3	Excluding those in patterns 5-6
	4	LHRH + anti-androgen > 3 months without prednisone or docetaxel	1	Including those who used both LHRH and anti-androgen between 60 days prior to prostate cancer diagnosis and 180 days following diagnosis (any drug might be initiated first)
			2	Including those who had continuous use of anti-androgen >3 months (with ≤14-day gap)
			3	Excluding those in patterns 5-6
ADT± anti-androgen + AA+P	5	LHRH +/- anti-androgen + prednisone[a]	1	Including those who initiated LHRH between 60 days prior to prostate cancer diagnosis and 180 days following diagnosis
			2	Including those who had continuous use of prednisone (5 mg PO or bid, supplies ≥ 90 days with ≤14-day gap) within 4 months of LHRH initiation
			3	Excluding those in pattern 6
ADT± anti-androgen + docetaxel	6	LHRH +/- anti-androgen + docetaxel	1	Including those who initiated LHRH between 60 days prior to prostate cancer diagnosis and 180 days following diagnosis
			2	Including those who used docetaxel within 4 months of LHRH initiation
non-ADT	7	None of LHRH, anti-androgen, docetaxel and prednisone	1	Including patients in mCSPC cohort but not in patterns 1-6

[a]Concurrent use of prednisone and abiraterone, i.e., prescription date + supplies within +/- 7 days of the 2 drugs: n= 8, 15% of the 52 patients). AA+P = abiraterone acetate plus prednisone; ADT = androgen deprivation therapy; LHRH = Luteinizing hormone-releasing hormone; mCSPC = metastatic castration-sensitive prostate cancer.

Supplementary Table 6. Definition of variables to describe baseline characteristics

Category	Description
Demographics	Age at index date (from RPDB)<br>Socioeconomic status at index date (neighborhood income quintile)<br>Geographic region at index date (LHIN, rural vs non-rural)
Prostate cancer characteristics	PSA (median, IQR) immediately prior to prostate cancer diagnosis (must be within 6 months prior to diagnosis; if none available prior to diagnosis, check for one within 2 months following diagnosis; if none available by these criteria, mark as missing)<br>Using LOINC codes LOINC 19197-3, 2857-1, 35741-8)<br>Stage (OCR - best_stage_grp)<br>Grade (Gleason score)
General medical care	Comorbidity – Charlson (CCI; 2 year look back using DAD and SDS data, including index)<br>Number of GP visits (spec = 00) in year prior to diagnosis (deduped on ikn servdate physnum)<br>Hospitalization (inpatient for any reason) in year prior to diagnosis (yes/no)<br>Ever resident of long-term care in year prior to diagnosis (yes/no)
Specific comorbidity	Diabetes diagnosis (ODD) (up to 2017)<br>Myocardial infarction in 5 years prior to diagnosis (OMID)<br>Since OMID only up to date until 2016, use OMID and DAD<br>Definition: either flagged in OMID and/or Main diagnosis hospitalization with ICD-10 I21 with no prior hospitalization for MI 1 yr prior<br>Cerebrovascular accident in 5 years prior to diagnosis (NACRS, DAD)<br>ICD-10: I63.x,167.81, I67.82<br>DAD – Most responsible diagnosis<br>NACRS – Main diagnosis<br>Congestive heart failure (CHF ICES cohort) (up to 2017)<br>COPD (COPD ICES cohort) (up to 2017)<br>Hypertension (Hyper ICES cohort) (up to 2017)<br>ED/hospitalization for arrhythmia in year prior to diagnosis (NACRS, DAD)<br>ICD-10: I44.x, I45.x, I47.x, I48.x, I49.x<br>DAD – Most responsible diagnosis<br>NACRS – Main diagnosis<br>Diagnosis of dementia within 5 years of diagnosis (NACRS, DAD, OHIP)<br>ICD-10: F01.x, F02.x, F03.x, G30.X, G31.83,<br>DXCODE: 290<br>DAD – Most responsible diagnosis<br>NACRS – Main diagnosis<br>OHIP - DXCODE<br>Diagnosis of liver disease within 5 yrs prior to diagnosis (NACRS, DAD, OHIP)<br>ICD-10: K70.x, K71.x, K72.x, K73.x, K74.x, K75.2, K75.3, K75.4, K75.8, K75.9, K76.9<br>DXCODE: 571<br>DAD – Most responsible diagnosis<br>NACRS – Main diagnosis<br>OHIP - DXCODE<br>Diagnosis of renal disease within 5 yrs prior to diagnosis (NACRS, DAD, OHIP)<br>ICD-10: N18.x, N19<br>DXCODE: 585<br>DAD – Most responsible diagnosis<br>NACRS – Main diagnosis<br>OHIP - DXCODE
